# Prospective study of factors influencing conditional discharge from a forensic hospital: the DUNDRUM-3 programme completion and DUNDRUM-4 recovery structured professional judgement instruments and risk

**DOI:** 10.1186/1471-244X-13-185

**Published:** 2013-07-09

**Authors:** Mary Davoren, Zareena Abidin, Leena Naughton, Olivia Gibbons, Andrea Nulty, Brenda Wright, Harry G Kennedy

**Affiliations:** 1 National Forensic Mental Health Service, Central Mental Hospital, Dundrum, Dublin 14, Ireland; 2 Department of Psychiatry, Trinity College, Dublin, Ireland

## Abstract

**Background:**

We set out to examine whether structured professional judgement instruments DUNDRUM-3 programme completion (D-3) and DUNDRUM-4 recovery (D-4) scales along with measures of risk, mental state and global function could distinguish between those forensic patients detained in a secure forensic hospital (not guilty by reason of insanity or unfit to stand trial) who were subsequently discharged by a mental health review board. We also examined the interaction between these measures and risk, need for therapeutic security and eventual conditional discharge.

**Methods:**

A naturalistic observational cohort study was carried out for 56 patients newly eligible for conditional discharge. Patients were rated using the D-3, D-4 and other scales including HCR-20, S-RAMM, START, SAPROF, PANSS and GAF and then observed over a period of twenty three months during which they were considered for conditional discharge by an independent Mental Health Review Board.

**Results:**

The D-3 distinguished which patients were subsequently discharged by the Mental Health Review board (AUC = 0.902, p < 0.001) as did the D-4 (AUC = 0.848, p < 0.001). Item to outcome analysis showed each item of the D-3 and D-4 scales performed significantly better than random. The HCR-20 also distinguished those later discharged (AUC = 0.838, p < 0.001) as did the S-RAMM, START, SAPROF, PANSS and GAF. The D-3 and D-4 scores remained significantly lower (better) for those discharged even when corrected for the HCR-20 total score. Item to outcome analyses and logistic regression analysis showed that the strongest antecedents of discharge were the GAF and the DUNDRUM-3 programme completion scores.

**Conclusions:**

Structured professional judgement instruments should improve the quality, consistency and transparency of clinical recommendations and decision making at mental health review boards. Further research is required to determine whether the DUNDRUM-3 programme completion and DUNDRUM-4 recovery instruments predict those who are or are not recalled or re-offend after conditional discharge.

## Background

The Butler Report [1] described conditional discharge as the “most valuable feature of the system of restriction orders” and it has been shown that patients who have been conditionally discharged from forensic hospitals have lower rates of recidivism than those released without conditions [2]. Coid et al. showed that longer stay and restriction on discharge are protective factors against reoffending in patients discharged from medium secure forensic psychiatry services [3]. However despite the benefits of conditional discharge, the decision to recommend a patient for conditional discharge is one of the most difficult taken by a forensic psychiatrist. Mental health tribunals and review boards make decisions that require the balancing of rights and risks according to law. Patients should not be detained at a level of therapeutic security higher than that which is deemed necessary, for their own safety and the safety of others [4] however this must be balanced with public safety and victim rights issues.

Risk assessment is a key part of the process when making decisions regarding a patient’s readiness for discharge to the community. Dolan and Khawja [5] showed that the HCR-20 was a predictor of readmission and self reported violence in groups of discharged male medium secure patients. It has also been shown that the HCR-20 is a good predictor of both violent and non-violent offending in patients released from forensic psychiatric hospitals [6]. Doyle at al have also shown that dynamic (“current” and “risk”) items on the HCR-20 significantly improved the accuracy of prediction of violence after discharge from forensic units [7]. It has been shown that factors such as a higher score on PCL-R and a younger age at the time of first criminal offence were significantly related to release recommendations in a group of NGRI patients in the USA [8]. However evidence presented to Criminal Law (Mental Health) Review boards, when recommending a patient for a move to less secure places, consists of more than risk assessment. In practice evidence given by clinicians also includes or takes account of factors such as rapport, insight, therapeutic alliance and use of leave from the hospital. Risk assessment in forensic psychiatry has evolved in recent years from the use of unstructured clinical judgement to the use of structured professional judgement instruments. These instruments add to the transparency and accountability of the process of risk assessment, uniting the tasks of prediction, assessment, clinical management and communication [9]. The aim of the DUNDRUM toolkit is to provide the same transparency and accountability to the decisions regarding admission to and discharge from secure hospital settings [10].

The DUNDRUM toolkit is a set of five structured professional judgement instruments developed at the Central Mental Hospital Dundrum, the Republic of Ireland’s only secure forensic hospital [10]. The first two instruments of the DUNDRUM Toolkit are concerned with the admission of patients to secure hospitals, acting as structured professional judgement instruments to decide the level of need for therapeutic security and the urgency of that need [10]. We have previously shown that these two instruments distinguished between patients who required admission to different levels of therapeutic security and distinguished the urgency of need amongst patients [11,12]. The DUNDRUM-3 and DUNDRUM-4 scales are structured professional judgement instruments to rate a patient’s programme completion and recovery and therefore the patient’s continuing need for therapeutic security [10]. We have also shown that the DUNDRUM-3 programme completion items distinguished significantly between levels of therapeutic security and the DUNDRUM-4 recovery items consistently distinguished those given unaccompanied leave outside the secure hospital setting [13]. The fifth scale is a self-rated instrument which patients use to rate their own recovery and their own ongoing need for therapeutic security and the validation of this instrument is currently underway. We have recently shown that the DUNDRUM-1 triage security scale and HCR-20 instrument for the assessment of risk of violence distinguished between those who were moved from higher to less secure wards within the hospital and also from less secure back to more secure wards, and the DUNDRUM-3 and DUNDRUM-4 also distinguished those who were moved [14]. Our hypothesis is that it should be possible to use structured professional judgement to guide decision making about conditional or absolute discharge from hospital.

The Criminal Law (Insanity) Act 2010 [15] was introduced in the Republic of Ireland in March 2011. It introduced, for the first time in Ireland, the power for mental health review boards (MHRBs) to grant conditional discharge to those detained following a verdict of not guilty by reason of insanity (NGRI) or unfit to stand trial (UST). This ’experiment of nature’ offered the opportunity to carry out a prospective observational study on the clinical facts that influence how clinicians make such recommendations and MHRBs make such decisions.

In this prospective cohort study we examined whether the DUNDRUM-3 programme completion and DUNDRUM-4 recovery instruments [10] measured at baseline just before the commencement of the new legislation could distinguish between those who were or were not subsequently discharged either conditionally or unconditionally from this high and medium secure forensic hospital setting, and whether measures of risk, need for therapeutic security, mental state and global function also played a part in the decision. Because we are interested in the use of structured professional judgement itself, we also wished to study the ‘item to outcome’ results for these instruments, since a study of the scale scores would be insufficient, amounting to an actuarial study only.

## Methods

### Study design

This was a naturalistic prospective observational cohort study. Data were gathered as part of the routine clinical audit of service delivery and outcomes. The study was approved by the research ethics, audit and effectiveness committee of the Central Mental Hospital. All participants were given information about the nature and purpose of the assessments and gave informed consent.

Structured professional judgement instruments and measures of mental state and global function were assessed at baseline in February 2011 just before the commencement of the Criminal Law (Insanity) Act 2010 which permitted conditional discharge for the first time. This allowed the MHRB to grant conditional discharge where appropriate and safe to do so, and subject to conditions regarding both treatment adherence and supports according to the individual’s risks and needs.

The Mental Health Review Board was supplied with a report from the treating consultant psychiatrist, had access to all clinical notes, assessments and reports and heard oral evidence from the patient, the treating consultant psychiatrist and the patient’s lawyer, and any other evidence the MHRB wished to hear. The clinicians and MHRB were blind to the assessments reported here, carried out independently of the treating clinicians prior to the commencement of the new legislation. However neither the clinicians nor the members of the MHRB could be blinded to the unstructured factual clinical information used to make the ratings for the various instruments, nor could they have been blinded to such information. Clinicians decided to recommend or not to recommend absolute or conditional discharge in accordance with their normal practice, a synthesis of the results of structured professional judgement instruments such as the HCR-20 (rated by themselves) and broad unstructured bio-psycho-social assessment including reports of progress in hospital. The MHRB received reports from the treating psychiatrists and other clinicians and was independent in the exercise of its powers [15].

### Setting

The Central Mental Hospital is the only secure forensic psychiatric hospital in the state, providing high, medium and low secure therapeutic wards for a population of 4.6 million. It is the only hospital designated under the Criminal Law (Insanity) Acts 2006 & 2010 for the reception and treatment of patients found unfit to stand trial or not guilty by reason of insanity, or transferred from prison to hospital for psychiatric treatment. Male patients in the Central Mental Hospital are admitted to a high secure admission ward. The hospital is organised into a series of progressively less secure wards and patients are moved from ward to ward along this recovery pathway, progressing from high secure admission units, through medium secure units and on to low secure pre-discharge rehabilitation units [16]. These placements correspond to a coherent patient-centred pathway through care as patients are placed at the level of therapeutic security deemed most appropriate for their needs. Placements also correspond to a stratified risk management system [17].

### Legal structures

Decisions to offer conditional or unconditional discharges to patients detained under the Criminal Law (Insanity) Acts 2006 & 2010 on the grounds of unfitness to stand trial (UST) or not guilty by reason of insanity (NGRI) were made by a Mental Health (Criminal Law) Review Board (MHRB). Those detained under the Mental Health Act 2001 were reviewed by a different body, the Mental Health Tribunal [18] that could grant absolute discharges but not conditional discharges or community treatment orders.

### Participants

All patients in the hospital were assessed between February and March 2011 of whom 56 were newly eligible for conditional discharge to the community by the mental health review board. Data on the eligible patients and their subsequent discharges from the hospital were then gathered up to 31st December 2012. Those detained because UST (8 patients) were eligible for conditional discharge by the MHRB in the same way as those detained because found NGRI (48 patients), though those detained because UST could also be returned to court by the treating consultant if they regained fitness.

### Variables

The primary outcome measure was absolute or conditional discharge by the MHRB from this high and medium secure forensic hospital setting following the commencement of the Criminal Law (Insanity) Act 2010 on 8th February 2011. Patient discharges were documented up to 31st December 2012, an observation period of twenty three months. During this period each of the 56 patients eligible for discharge had at least three Criminal Law Review Board hearings thus ensuring that each patient had the opportunity to be granted a conditional or absolute discharge.

### Data sources and measurement

The DUNDRUM-1 triage security instrument, DUNDRUM-3 programme completion (D-3) and DUNDRUM-4 recovery (D-4) scales are part of a set of four structured professional judgement instruments for the assessment of need for therapeutic security [10]. The DUNDRUM-1 triage security scale is a static measure while the DUNDRUM-3 and DUNDRUM-4 are dynamic measures sensitive to change and response to treatment. The content of these instruments is different from but complementary to risk assessment. When assessing readiness for discharge to the community, clinicians are likely to take more than risk assessment alone into account. Factors such as mental health, physical health, self care and activities of daily living, family and social networks, use of leave from the hospital and other such factors are all given strong consideration. These items are often included in clinician’s unstructured reports to mental health tribunals and review boards to assist these bodies in their decision making with regard to a patient’s readiness for conditional or absolute discharge, or neither. The items scored in the DUNDRUM-3 and DUNDRUM-4 include the above items and the item definitions and ratings are based on motivation theory, cycle of change and engagement. The DUNDRUM-3 programme completion instrument consists of seven items - physical health, mental health, drugs and alcohol, problem behaviours, self care and activities of daily living, occupation, education and creativity and family and social networks. The six items of the DUNDRUM-4 recovery scale are stability, insight, rapport and working alliance, leave, HCR-20 dynamic risk items and victim sensitivity. Each item is accompanied by a series of definitions and rated from ‘0’ to ‘4’. A patient scoring mostly ‘4’s is unlikely to be ready for a move to a less secure place, a patient scoring mainly ‘3’s is likely to be ready to move from high to medium security, mainly ‘2’s is likely to be ready to move from medium security to PICU, mainly ‘1′s is ready for placement in the community setting and mainly ‘0’s is likely to be ready for an absolute discharge.

We have previously shown that the DUNDRUM-3 programme completion and the DUNDRUM-4 recovery scales have excellent psychometric properties [13] and distinguished between patients who moved between levels of therapeutic security within the forensic hospital setting [14] while the DUNDRUM-1 triage security scale and the HCR-20 risk assessment score [19] accounted for most of the variation in this decision [14].

As part of the hospital’s routine data gathering for audit and outcome measures, all in-patients were assessed at baseline prior to the commencement of the Criminal Law (Insanity) Act 2010 by MD using the DUNDRUM-1 triage security instrument, DUNDRUM-3 programme completion instrument and DUNDRUM-4 recovery instrument between February and March 2011. All patients were also assessed for the Positive and Negative Symptom Score (PANSS) [20] and Global Assessment of Function (GAF) [21] by post membership psychiatrists (OG and LN) who were blind to the ratings of DUNDRUM-3 and DUNDRUM-4. The HCR-20 [19] and S-RAMM [22] were rated by the treating clinicians and collated by AN. The HCR-20 historical item H7 ‘psychopathy’ was omitted. ZA rated all patients using the START [23] and the Structured Assessment of Protective Factors for Violence Risk (SAPROF) [24]. The treating clinicians were blind to these audit and outcome ratings when making their reports to the MHRB, as were the MHRB.

### Confounding and bias

Possible sources of confounding and bias were considered, including the need for therapeutic security at baseline measured by the DUNDRUM-1 triage security instrument [10] since historical considerations might influence clinicians to be more conservative when recommending whether a patient be offered a conditional or unconditional discharge, or neither. The DUNDRUM-1 was also used to enable benchmarking against other services and other studies. Other confounders considered included a measure of risk of violence the HCR-20 [19], risk of suicide and self harm measured by the S-RAMM [22] and protective factors the START-S [23] and SAPROF [24], a measure of mental state (PANSS) [20] and global function (GAF) [21].

### Statistical methods

No data were missing. All data were entered in SPSS-18 [25].

Association with outcome was tested using the receiver operating characteristic (ROC) area under the curve (AUC), where a significant result for the AUC is one that differs significantly from the ‘random’ AUC of 0.5 - as a minimum the 95% confidence interval for the AUC does not overlap 0.5.

Where analysis of variance was carried out, differences between groups can be assessed from the over-lap of confidence intervals. Univariate analysis of a general linear model was carried out for discharge, co-varying for the HCR-20 total score at the beginning of the period of observation because the HCR-20 measure of dynamic risk is already established as a predictor of success or failure in such discharges [5-8]. Similarly, we tested whether the HCR-20 scores differentiated between those discharged and those not when co-varying for DUNDRUM-3 and DUNDRUM-4 scores.

Regression analysis was used to examine the antecedent covariates of discharge. Discharge was treated as a binary variable, and binary logistic regression was used with either forward or backward stepwise likelihood ratios as appropriate.

Covariates were chosen according to the results of the receiver operating characteristic.

Unadjusted odds ratios were used as an indicator of the magnitude of effect of individual items, where these could be calculated.

## Results

### Participants

There were 56 eligible patients. The mean follow up period was 1.748 years (SD 0.248). Twelve were conditionally discharged and none were absolutely discharged as a first outcome during the follow up period (Table 1). One who had been conditionally discharged was subsequently absolutely discharged – this was not double counted.

**Table 1:** Receiver operating characteristic area under the curve (AUC), with 95% confidence intervals and asymptotic ‘p’ value for null hypothesis that AUC does not differ from 0.5, for 56 patients of whom 12 were granted a conditional discharge

	**Area under the curve**	**95% confidence interval of area under the curve**		**p**	**Odds Ratio**	**95% confidence interval of odds ratio**		**p**
		**Lower**	**Upper**			**Lower**	**Upper**	
DUNDRUM-1 triage security	0.624	0.446	0.802	0.192	0.915	0.767	1.078	0.286
DUNDRUM-3 programme completion	0.902	0.808	0.995	<0.001	0.695	0.561	0.861	0.001
DUNDRUM-4 recovery	0.848	0.742	0.953	<0.001	0.761	0.638	0.908	0.002
HCR-H historical	0.745	0.584	0.906	0.010	0.690	0.523	0.910	0.009
HCR-C clinical	0.820	0.691	0.949	0.001	0.513	0.319	0.824	0.006
HCR-R risk	0.663	0.497	0.828	0.086	0.673	0.427	1.060	0.087
HCR-dynamic (C + R)	0.788	0.644	0.931	0.002	0.694	0.518	0.929	0.014
HCR-total	0.838	0.710	0.966	<0.001	0.728	0.586	0.904	0.004
S-RAMM-B background	0.546	0.358	0.734	0.633	0.948	0.748	1.202	0.660
S-RAMM-C current	0.765	0.613	0.917	0.006	0.538	0.325	0.892	0.016
S-RAMM-F future	0.680	0.498	0.862	0.060	0.766	0.586	1.002	0.052
S-RAMM dynamic (C + F)	0.738	0.583	0.892	0.013	0.798	0.665	0.958	0.016
S-RAMM total	0.869	0.525	0.859	0.045	0.848	0.736	0.977	0.022
SAPRFOF	0.806	0.654	0.958	0.001	1.305	1.059	1.607	0.013
START-V	0.899	0.789	1.000	<0.001	1.430	1.135	1.803	0.002
START-S	0.904	0.755	1.000	<0.001	0.625	0.461	0.847	0.002
GAF	0.930	0.845	1.000	<0.001	1.259	1.090	1.454	0.002
PANSSpos	0.758	0.625	0.891	0.007	0.709	0.478	1.050	0.086
PANSSneg	0.809	0.693	0.925	0.001	0.739	0.557	0.982	0.037
PANSSgen	0.809	0.686	0.932	0.001	0.790	0.659	0.946	0.010
PANSStotal	0.846	0.736	0.955	<0.001	0.873	0.778	0.978	0.020

Note that lower scores are calculated as positive predictors of discharge, yielding higher AUCs, except for GAF, SAPROF and START-S, where higher scores are positive predictors yielding higher AUCs. Unadjusted odds ratios (OR) with 95% confidence intervals and Wald p value.

### Descriptive data

At baseline, mean age was 43.7 years (SD 12.8) and mean length of stay was 11.1 years (SD 11.3) for the 56 eligible patients. All were Irish born. There were no significant differences between those discharged by the MHRB (n = 12) and those who were not discharged (n = 44) though there was a tendency for those conditionally discharged to be older (44.1(10.0) years vs 42.3(13.6)) and to have had longer lengths of stay (11.6(8.7) years vs 10.9(11.9)). Diagnosis according to ICD-10 criteria [26] was schizophrenia (F20) 41 (73%), schizoaffective disorder (F25) 4 (7%), bi-polar affective disorder (F31) 5 (9%), recurrent depressive disorder, severe with psychotic symptoms (F33.3) 4 (7%), intellectual disability (mental retardation with significant impairment of behaviour F71.1 and 72.1) 2 (4%). The legal status was unfit to stand trial 8 (14%), not guilty by reason of insanity 48 (86%).

The need for therapeutic security was assessed at baseline using the DUNDRUM-1 triage security scale [10]. The mean DUNDRUM-1 score for the 56 eligible participants was 28.6 (SD 4.2). As there are 11 items in the D-1 scale all scored from ‘0’ to ‘4’ this equates to a mean item score of 2.6, where ‘2’ would be a score consistent with a low secure profile and ‘3’ would be a score consistent with a medium secure profile [15], the mean for all was therefore higher than the level of a ‘typical’ low secure patient.

### Outcome data

Of the 8 patients admitted because they were deemed unfit to stand trial 5 remained as in-patients during the period of observation while three were found fit to stand trial in court and returned to prison or the community. All had been considered by the Mental Health Review Board but had not been discharged. Of the 48 patients who had the legal status of Not Guilty by Reason of Insanity (NGRI) 12 were discharged subject to conditions by the MHRB while the remaining 36 continued to be detained as in-patients in hospital at the end of the period of observation.

### Main results

#### Receiver operating characteristics

For the 56 eligible for conditional or absolute discharge by the MHRB, Table 1 shows the area under the curve (AUC) of the receiver operating characteristic with discharge (all conditional) by the MHRB as the outcome measure.

Neither length of stay at baseline (AUC = 0.598, 96% CI 0.434 – 0.763, p = 0.299) nor age at baseline (AUC = 0.558, 95% CI 0.393 – 0.722, p = 0.539) was associated with subsequent discharge.

The DUNDRUM-1 triage security scale was not associated with subsequent conditional discharge (AUC = 0.624, 95% CI 0.446 – 0.802, p = 0.192). The DUNDRUM-3 Programme completion scale distinguished which patients went on to receive a discharge from the Criminal Law (Mental Health) Review board (AUC = 0.902, p < 0.001) as did the DUNDRUM-4 Recovery Scale (AUC = 0.848, p < 0.001).

The HCR-20 total score also distinguished which patients went on to receive a discharge (AUC = 0.838, p < 0.001) as did the HCR-20 historical items (AUC = 0.745, p = 0.010) and HCR-20 dynamic (clinical and risk) items (AUC = 0.788, p = 0.002).

The S-RAMM background items did not distinguish which patients went on to receive a discharge from the Criminal Law (Mental Health) Review board (AUC 0.546, p = 0.633), however the S-RAMM dynamic (current and future) items (AUC = 0.738, p = 0.013) and S-RAMM total score (AUC = 0.869, p = 0.045) were significant.

The START strengths distinguished which patients went on to receive a conditional discharge (AUC = 0.904, p < 0.001) as did the START vulnerabilities (AUC = 0.899, p < 0.001) and the SAPROF (AUC = 0.806, p = 0.001).

The Global Assessment of Functioning (GAF) Scale distinguished which patients received a discharge from the Criminal Law (Mental Health) Review board (AUC = 0.930, p < 0.001).

The PANSS positive scale distinguished which patients went on to receive a discharge (AUC = 0.758, p = 0.007) as did the PANSS negative scale (AUC = 0.809, p = 0.001), PANSS general symptoms score (AUC = 0.809. p = 0.001) and the total PANSS score (AUC = 0.846, p < 0.001).

#### Analysis of variance

Table 2 shows that the DUNDRUM-3 and DUNDRUM-4 scores, including the DUNDRUM-4 score omitting the item for ‘risk’ were all significantly lower (better) at baseline for those who went on to be conditionally discharged. The GAF score was significantly higher (better) and PANSS positive, negative and general scores were significantly lower (better) at baseline for those who were later discharged. The HCR-20 historical, clinical, risk and total scores, S-RAMM current and future scores were lower (better) as was the START-V score, while the SAPROF and START-S were higher (better) in those who were later discharged. When co-varied for HCR-20 total score, only the DUNDRUM-3 and DUNDRUM-4 scores remained significantly better at baseline in those who went on to be discharged, though the GAF and START scores neared statistical significance.

**Table 2:** Determinants of discharge: crude data and marginal means adjusted for HCR-20 total score

	**Crude data, means (SD)**			**Marginal means (SE) adjusted for HCR-20 total scores**		
	**Not discharged**	**Discharged**	**ANOVA**	**Not discharged**	**Discharged**	**ANOVA**
	**N = 44**	**N = 12**	**F/p**	**N = 44**	**N = 12**	**F/p**
DUNDRUM-1 triage security	28.9(4.4)	27.4(3.2)	1.1/0.290	28.4(0.6)	29.0(1.2)	0.2/0.656
DUNDRUM-3 programme completion	13.9(6.7)	3.9(4.6)	14.6/0.001	13.7(0.7)	8.3(1.5)	10.1/0.002
DUNDRUM-4 recovery	13.8(6.1)	4.4(3.5)	15.3/0.001	13.3(0.7)	10.0(1.4)	6.6/0.013
DUNDRUM-4 omitting item 5 ‘risk’	11.7(4.7)	5.9(5.0)	9.2/0.004	11.6(0.6)	9.7(1.3)	1.8/0.2
GAF	49.7(15.0)	70.0(9.6)	11.9/0.001	49.6(1.6)	62.0(3.2)	3.9/0.051
**PANSS**						
Positive	14.1(9.0)	7.5(1.7)	6.3/0.015	13.2(1.1)	11.1(2.2)	0.1/.9
Negative	17.7(10.0)	8.7(2.0)	9.7/0.003	16.8(1.2)	12.3(2.5)	0.6/0.5
General	31.6(12.9)	20.0(3.5)	9.3/0.004	30.2(1.6)	25.2(3.2)	0.6/0.5
Total	63.5(29.4)	36.2(6.5)	10.0/0.002	60.1(3.5)	48.6(7.1)	0.4/0.5
**HCR-20**						
Historical	12.5(3.9)	9.5(2.9)	5.8/0.019	n/a	n/a	n/a
Clinical (current)	3.6(2.8)	1.0(1.2)	9.9/0.003	n/a	n/a	n/a
Risk (future)	2.7(1.9)	0.5(0.5)	13.9/<0.001	n/a	n/a	n/a
Dynamic (C + R)	6.2(4.3)	1.5(1.5)	13.5/0.001	n/a	n/a	n/a
Total	18.7(6.3)	11.0(3.8)	16.1/<0.001	n/a	n/a	n/a
**S-RAMM**						
Background	9.4(2.8)	9.0(2.9)	0.2/0.667	9.2(0.4)	9.6(0.9)	0.09/0.759
Current	4.5(2.6)	2.33(1.5)	7.3/0.009	4.2(0.3)	3.4(0.7)	0.96/0.331
Future	5.2(2.6)	3.4(2.7)	4.2/0.044	4.9(0.4)	4.5(0.7)	0.18/0.672
Dynamic (C + F)	9.6(4.6)	5.8(3.7)	7.2/0.010	9.0(0.6)	7.9(1.2)	0.66/0.421
Total	19.0(5.3)	14.8(4.9)	6.23/0.16	18.3(0.7)	17.4(1.4)	0.26/0.612
SAPROF	23.3(6.1)	28.8(4.2)	8.52/0.005	24.3(0.7)	25.3(1.3)	0.4/0.515
START-S	27.84(8.7)	38.16(4.0)	15.7/<0.001	29.1(0.9)	33.2(1.8)	3.6/0.060
START-V	10.8(7.6)	1.8(3.1)	16.0/<0.001	9.7(0.8)	6.1(1.6)	3.9/0.054

Table 3 shows that when the HCR-20 sub-scales and total scores were adjusted for DUNDRUM-3 or DUNDRUM-4 (omitting the risk item from the DUNDRUM-4) they were no longer significantly better for those conditionally discharged.

**Table 3:** HCR-20 scores and discharge by tribunals - crude data and marginal means adjusted for the DUNDRUM-3 programme completion score and DUNDRUM-4 recovery score (omitting item D-4-R item 5 ′risk’ to avoid circularity)

	**‘Raw’ data, means (S.D.)**			**Marginal means (S.E.) adjusted for DUNDRUM-3 score**			**Marginal means (S.E.) adjusted for DUNDRUM-4 score (omitting item 5 ‘risk’)**		
	**Not discharged**	**Discharged**	**ANOVA**	**Not discharged**	**Discharged**	**ANOVA**	**Not discharged**	**Discharged**	**ANOVA**
HCR-20	N = 44	N = 12	F/p	N = 44	N = 12	F/p	N = 44	N = 12	F/p
Historical	12.5(3.9)	9.5(2.9)	5.8/0.019	11.7(0.4)	10.5(0.8)	1.4/0.2	11.8(0.4)	10.0(0.8)	3.3/0.074
Clinical	3.6(2.8)	1.0(1.2)	9.9/0.003	3.5(0.3)	3.4(0.6)	0.007/0.9	3.6(0.3)	3.1(0.6)	0.4/0.5
Risk	2.7(1.9)	0.5(0.5)	13.9/<0.001	2.5(0.3)	3.3(0.6)	1.2/0.3	2.5(0.3)	3.2(0.6)	1.3/0.3
Dynamic	6.2(4.3)	1.5(1.5)	13.5/0.001	6.0(0.5)	6.8(1.4)	0.3/0.6	6.1(0.5)	6.4(1.0)	0.7/0.8
Total	18.7(6.3)	11.0(3.8)	16.1/<0.001	17.1(0.6)	16.4(1.4)	0.2/0.7	17.2(0.6)	16.0(1.2)	0.8/0.4

#### Logistic regression

Binary logistic regression was performed using forward stepwise likelihood ratios for the variables with significant areas under the curve. These were the DUNDRUM-3, DUMDRUM-4, HCR-20 total score, S-RAMM current score, START-S, SAPROF, GAF and PANSS total scores. The START-V was omitted and HCR-20 sub-scales combined to reduce multiple co-linearity while the START-S was also omitted to avoid co-linearity with SAPROF. The iterative process resolved in one step for the GAF as the only significant variable remaining in the regression equation. The model was acceptable with Hosmer and Lemeshow X^2^ = 8.45, df = 8, p = 0.391, Nagelkerke R^2^ = 0.610, and GAF odds ratio = 1.258 (95% confidence interval 1.089 – 1.454) p = 0.002. This model correctly distinguished 95.1% of those not discharged, 83.1% of those who were discharged and 92.5% overall. This model was not robust with backwards logistic regression yielding a different result, resolving in five steps (DUNDRUM-3 odds ratio = 0.682, 95% CI 0.470 – 0.991, p = 0.045; SAPROF OR = 0.633, 95% CI 0.413 – 0.967, p = 0.034; GAF OR = 1.387, 95% CI 1.054 – 1.825, p = 0.020, Hosmer and Lemeshow X^2^ = 3.629, df = 8, p = 0.889, Nagelkerke R^2^ = 0.739, correct predictions as before).

Because the model was not robust, the logistic regression was repeated, this time omitting the GAF. This was done because of the apparent interactive effects between the GAF and SAPROF, evident also when the START-S was included instead of the SAPROF. Backward logistic regression resolved in five iterations, Nagelkerke R^2^ = 0.603, Hosmer and Lemeshow X^2^ = 6.05, df = 8, p = 0.642, with 97.6% correct for those not discharged, 66.7% correct for those discharged and 90.6% correct overall. The model included two variables DUNDRUM-3 OR = 0.717, 95% CI 0.561 – 0.916, p = 0.008 and PANSS total score which did not reach significance OR = 0.913, 95% CI 0.821 – 1.016, p = 0.097. This model was reasonably robust and when repeated by forward logistic regression yielded a model in one iteration with Nagelkerke R^2^ = 0.525, Hosmer and Lemeshow X^2^ = 2.37, df = 7, p = 0.927, 92% correct for those not discharged, 66.7% correct for those discharged and 86.8% correct overall and included a single variable, DUNDRUM-3 odds ratio = 0.698, 95% CI 0.563 – 0.866, p < 0.001.

#### Secondary analysis

On an item to outcome analysis, all items of the DUNDRUM-3 programme completion scale and all items of the DUNDRUM-4 recovery scale were significantly related to eventual discharge (Table 4). Although the DUNDRUM-1 triage security scale was not a significant indicator of subsequent conditional discharge, a lower score on item TS7 ‘preventing access’ was associated with conditional discharge (AUC = 0.726, 95% CI 0.539 – 0.912, p = 0.018) and a lower score on a further item, TS5 ′specialist forensic need’ was strongly associated with subsequent conditional discharge (AUC = 0.845, 95% CI 0.725 - 0.966, p = 0.003).

**Table 4:** Item to outcome measures for DUNDRUM-3 programme completion and DUNDRUM-4 recovery

	**AUC**	**95% confidence interval of AUC**		**p**	**Odds ratio**	**95% confidence interval of odds ratio**		**p**
**DUNDRUM-3 programme completion**		**Lower**	**Upper**			**Lower**	**Upper**	
PC1 (Physical health)	0.856	0.754	0.958	<0.001	0.169	0.054	0.528	0.002
PC2 (Mental health)	0.855	0.754	0.957	<0.001	0.139	0.035	0.551	0.005
PC3 (Drugs and alcohol)	0.742	0.605	0.880	0.011	0.364	0.158	0.840	0.018
PC4 (Problem behaviours)	0.865	0.763	0.966	<0.001	0.302	0.147	0.619	0.001
PC5 (Self care and activities of daily living)	0.881	0.788	0.973	<0.001	0.100	0.024	0.417	0.002
PC6 (Education, occupation and creativity)	0.850	0.743	0.958	<0.001	0.115	0.030	0.440	0.002
PC7 (Family and social networks)	0.697	0.511	0.883	0.038	0.564	0.318	0.999	0.050
**DUNDRUM-4 recovery**								
R1 (Stability)	0.851	0.745	0.957	<0.001	0.137	0.033	0.559	0.006
R2 (Insight)	0.868	0.766	0.970	<0.001	0.068	0.009	0.493	0.008
R3 (Therapeutic rapport)	0.720	0.544	0.895	0.021	0.466	0.234	0.929	0.030
R4 (Leave)	0.711	0.571	0.852	0.026	0.466	0.232	0.939	0.033
R5 (Dynamic risk items)	0.805	0.687	0.923	0.001	0.308	0.136	0.702	0.005
R6 (Victim sensitivity)	0.748	0.586	0.910	0.009	0.500	0.295	0.849	0.010

Note that lower (better) scores are calculated to be positive predictors of discharge yielding higher AUCs. *AUC* area under the curve (receiver operating characteristic). ‘p’ = Asymptotic probability for null hypothesis that AUC = 0.5. Unadjusted odds ratios (OR) with 95% confidence intervals and Wald p values.

For the HCR-20 historical (fixed) items only low scores on H3 ‘relationship instability’ and H4 ‘employment problems’ distinguished subsequent discharge, though H7 psychopathy was omitted. Amongst the dynamic items the associations of subsequent discharge were lower scores for C1 ‘lack of insight’, C3 ‘active symptoms of major mental illness’, C5 ‘unresponsiveness to treatment’ and R1 ‘plans lack feasibility’ (Table 5).

**Table 5:** Item to outcome measures for HCR-20

	**AUC**	**95% confidence interval**		**p**	**Odds ratio (OR)**	**95% confidence interval for OR**		**p**
	**AUC**	**Lower**	**Upper**			**Lower**	**Upper**	
**HCR-20 Historical items**								
H1 (Previous violence)	0.500	0.314	0.686	1.000	0.261	-	-	0.001
H2 (Young age at first violent incident)	0.553	0.361	0.746	0.575	0.684	0.227	2.062	0.500
H3 (Relationship instability)	0.749	0.612	0.886	0.009	0.337	0.137	0.831	0.018
H4 (Employment problems)	0.745	0.598	0.892	0.010	0.294	0.115	0.747	0.010
H5 (Substance use)	0.555	0.372	0.739	0.561	0.794	0.386	1.652	0.544
H6 (Major mental illness)	0.500	0.314	0.686	1.000	0.261			0.001
H7 (Psychopathy)	-	-	-	-				
H8 (Early maladjustment)	0.634	0.453	0.815	0.160	0.531	0.233	1.211	0.132
H9 (Personality disorder)	0.549	0.368	0.731	0.603	0.622	0.146	2.651	0.521
H10 (Prior supervision failure)	0.667	0.470	0.863	0.080	0.403	0.170	0.957	0.039
**HCR-20 Clinical items**								
C1 (Lack of insight)	0.779	0.625	0.934	0.003	0.147	0.042	0.513	0.003
C2 (Negative attitudes)	0.645	0.488	0.802	0.127	0.193	0.027	1.398	0.104
C3 (Active symptoms of major mental illness)	0.722	0.585	0.858	0.019	0.236	0.066	0.837	0.025
C4 (Impulsivity)	0.576	0.405	0.746	0.424	0.341	0.053	2.189	0.257
C5 (Unresponsiveness to treatment)	0.760	0.600	0.921	0.006	0.195	0.058	0.653	0.008
**HCR-20 Risk items**								
R1 (Plans lack feasibility)	0.719	0.580	0.857	0.021	0.139	0.020	0.940	0.043
R2 (Exposure to destabilisers)	0.548	0.373	0.723	0.611	0.602	0.186	1.942	0.395
R3 (Lack of personal support)	0.628	0.463	0.793	0.178	0.368	0.103	1.317	0.124
R4 (Non-compliance with remediation attempts).	0.485	0.303	0.666	0.873	0.920	0.316	2.683	0.879
R5 (Stress).	0.571	0.387	0.755	0.454	0.644	0.229	1.813	0.405

Note that lower scores are calculated as positive predictors of discharge yielding higher AUCs. *AUC* area under the curve (receiver operating characteristic). ‘p’ = Asymptotic probability for null hypothesis that AUC = 0.5. Note that H7 ‘psychopathy’ was omitted. Unadjusted odds ratios (OR) with 95% confidence intervals and Wald p values. Note that OR confidence intervals could not be calculated for HCR-H1 or HCR-H6.

For the SAPROF, the items that distinguished those later discharged were item 4 ‘coping’, item 5 ′self control’, item 6 ‘work’, item 8 ‘financial management’, item 11 ‘life goals’ and item 13 ‘social network’ (Table 6).

**Table 6:** SAPROF and conditional discharge

	**AUC**	**95% confidence interval for AUC**		**p**	**Odds Ratio (OR)**	**95% confidence interval for OR**		**P**
		**Lower**	**Upper**			**Lower**	**Upper**	
SO1. Intelligence	0.629	0.437	0.821	0.175	3.194	0.845	12.08	0.087
SO2. Secure attachment in childhood	0.602	0.469	0.766	0.281				0.998
SO3. Empathy	0.636	0.462	0.811	0.151	2.151	0.792	5.84	0.133
SO4. Coping	0.721	0.566	0.875	0.020	4.475	1.350	14.83	0.014
SO5. Self-control	0.686	0.531	0.840	0.050	4.990	1.055	23.61	0.043
SO6. Work	0.678	0.520	0.836	0.061	3.195	0.916	11.14	0.068
SO7. Leisure activities	0.643	0.478	0.808	0.132	2.698	0.740	9.83	0.133
SO8. Financial Management	0.705	0.565	0.844	0.031				0.998
SO9. Motivation for treatment	0.668	0.499	0.836	0.077	2.911	0.845	10.03	0.090
SO10. Attitudes towards authority	0.562	0.383	0.740	0.516	1.501	0.496	4.541	0.472
SO11. Life goals	0.786	0.662	0.910	0.003	13.518	1.727	105.81	0.013
SO12. Medication	0.579	0.390	0.768	0.407	1.386	0.522	3.68	0.513
SO13. Social network	0.761	0.637	0.886	0.006				0.998
SO14. Intimate relationship	0.561	0.366	0.755	0.523	2.336	0.622	8.77	0.209
SO15. Professional care	0.458	0.265	0.651	0.660				1.000
SO16. Living circumstances	0.458	0.265	0.651	0.660				1.000
SO17. External control	0.458	0.265	0.651	0.660				1.000

Note that higher scores are calculated as predictive of discharge and yield higher AUCs. Unadjusted odds ratios (OR) with 95% confidence intervals and Wald p value. Note that odds ratios could not be calculated for some items.

For START strengths, the significant items for subsequent discharge were higher scores on item 2 ‘relationships’, item 3 ‘occupational’, item 6 ‘mental state’, item 10 ‘external triggers‘, item 17 ‘insight’, item 18 ‘plans’, item 19 ‘coping’ and item 20 ’treatability‘ (Tables 7 and 8).

**Table 7:** START and conditional discharge

	**START strengths**				**START vulnerabilities**			
		**95% confidence interval**				**95% confidence interval**		
	**AUC**	**Lower**	**Upper**	**P**	**AUC**	**Lower**	**Upper**	**P**
ST1. Social skills	0.678	0.524	0.832	0.061	0.723	0.577	0.870	0.018
ST2. Relationships	0.818	0.701	0.936	0.001	0.736	0.580	0.892	0.013
ST3. Occupational	0.696	0.552	0.840	0.039	0.727	0.594	0.861	0.017
ST4. Recreational	0.638	0.481	0.796	0.145	0.661	0.507	0.815	0.090
ST5. Self-care	0.659	0.509	0.810	0.093	0.636	0.481	0.792	0.151
ST6. Mental state	0.828	0.711	0.945	0.001	0.739	0.608	0.869	0.012
ST7. Emotional state	0.678	0.527	0.829	0.061	0.675	0.522	0.829	0.065
ST8. Substance use	0.659	0.509	0.810	0.093	0.648	0.495	0.801	0.119
ST9. Impulse control	0.618	0.448	0.789	0.212	0.610	0.446	0.774	0.247
ST10. External triggers	0.714	0.572	0.856	0.024	0.641	0.548	0.802	0.137
ST11. Social support	0.657	0.501	0.813	0.097	0.727	0.594	0.861	0.017
ST12. Material resources	0.659	0.509	0.810	0.093	0.648	0.495	0.801	0.119
ST13. Attitudes	0.633	0.459	0.806	0.162	0.533	0.348	0.718	0.727
ST14. Medication adherence	0.659	0.509	0.810	0.093	0.545	0.369	0.722	0.632
ST15. Rule adherence	0.539	0.360	0.717	0.682	0.539	0.360	0.717	0.682
ST16. Conduct	0.609	0.444	0.774	0.251	0.545	0.369	0.722	0.632
ST17. Insight	0.873	0.766	0.980	<0.001	0.835	0.717	0.954	<0.001
ST18. Plans	0.773	0.652	0.894	0.004	0.795	0.681	0.910	0.002
ST19. Coping	0.830	0.725	0.934	0.001	0.788	0.650	0.926	0.002
ST20. Treatability	0.746	0.591	0.901	0.009	0.763	0.634	0.892	0.006

Note that for START-S strengths, higher values are calculated as predictive of discharge and yield higher AUCs. For START-V vulnerabilities, lower scores are calculated as predictive of discharge and yield higher AUCs.

**Table 8:** START and conditional discharge

	**START strengths**				**START vulnerabilities**			
		**95% confidence interval**				**95% confidence interval**		
	**OR**	**Lower**	**Upper**	**P**	**OR**	**Lower**	**Upper**	**P**
ST1. Social skills	4.395	0.984	19.623	0.052	0.161	0.034	0.757	0.021
ST2. Relationships	20.214	2.443	167.249	0.005	0.184	0.041	0.823	0.027
ST3. Occupational	6.018	0.940	38.513	0.058				0.998
ST4. Recreational	3.095	0.812	11.789	0.098	0.280	0.070	1.112	0.070
ST5. Self-care				0.998				0.999
ST6. Mental state	24.341	2.882	205.544	0.003				0.998
ST7. Emotional state	7.150	0.915	55.871	0.061	0.124	0.015	1.033	0.054
ST8. Substance use				0.998				0.998
ST9. Impulse control	2.354	0.587	9.445	0.227	0.250	0.035	1.769	0.165
ST10. External triggers	8.766	1.133	67.808	0.038	0.409	0.142	1.176	0.097
ST11. Social support	3.677	0.874	15.467	0.076				0.998
ST12. Material resources				0.998				0.998
ST13. Attitudes	2.346	0.674	8.161	0.180	0.917	0.259	3.245	0.893
ST14. Medication adherence				0.998				0.999
ST15. Rule adherence	2.102	0.272	16.249	0.476	0.476	0.062	3.677	0.476
ST16. Conduct	4.128	0.554	30.744	0.166				0.999
ST17. Insight	22.145	4.042	121.320	0.001	0.069	0.014	0.353	0.001
ST18. Plans				0.997				0.997
ST19. Coping				0.997	0.083	0.016	0.427	0.003
ST20. Treatability	5.923	1.305	26.896	0.021	0.091	0.012	0.688	0.020

Note that some odds ratios could not be calculated.

For START vulnerabilities the items that distinguished those not subsequently discharged were item 1 ‘social skills’, item 2 ‘relationships’, item 3 ‘occupational’, item 6 ‘mental state’, item 11 ‘social support’, item 17 ‘insight’, item 18 ‘plans’, item 19 ‘coping’ and item 20 ‘treatability’ (Tables 7 and 8).

For the S-RAMM, an assessment of risk of suicide and self harm, the items that distinguished those later discharged were lower scores for item C4 ‘treatment adherence’, C7 ‘psychosocial stress’, F2 ‘future service contact’, F3 ‘future response to drug treatment’ and F4 ‘future response to psychological interventions’ (Table 9).

**Table 9:** S-RAMM items related to outcomes

**S-RAMM items**	**AUC**	**95% confidence interval of AUC**		**p**	**Odds ratio**	**95% confidence interval of OR**		**p**
		**Lower**	**Upper**			**Lower**	**Upper**	
B1 History of deliberate self harm	0.474	0.275	0.672	0.783	1.186	0.582	2.416	0.639
B2 Seriousness of previous suicidality	0.411	0.215	0.607	0.349	1.513	0.724	3.159	0.271
B3 Previous hospitalisation	0.630	0.463	0.796	0.172	0.540	0.225	1.298	0.168
B4 Mental disorder	0.488	0.304	0.672	0.903	1.286	1.115	1.483	1.000
B5 Substance abuse disorder	0.554	0.372	0.737	0.568	0.792	0.368	1.703	0.550
B6 Personality	0.512	0.328	0.696	0.903	0.862	0.243	3.055	0.819
B7 Childhood adversity	0.529	0.370	0.688	0.760	0.906	0.423	1.939	0.799
B8 Suicide in the family	0.437	0.245	0.629	0.508	1.504	0.546	4.143	0.430
B9 Age, Gender and marital status	0.628	0.430	0.826	0.179	0.220	0.059	0.820	0.024
C1 Suicidal ideation, communication and intent	0.512	0.328	0.696	0.903	0.778	0.674	0.897	0.036
C2 Hopelessness	0.581	0.412	0.750	0.392				
C3 Psychological symptoms	0.650	0.486	0.815	0.114	0.452	0.178	1.149	0.095
C4 Treatment adherence	0.686	0.541	0.831	0.050				
C5 Substance use	0.500	0.314	0.686	1.000				
C6 Psychiatric admission and discharge	0.489	0.289	0.960	0.911	1.561	′0.089	27.253	0.760
C7 Psychosocial stress	0.709	0.534	0.884	0.028	0.254	0.073	0.882	0.031
C8 Problem solving deficits	0.682	0.513	0.852	0.055	0.349	0.125	0.974	0.045
F1 Access to preferred method of suicide	0.418	0.231	0.604	0.386	1.556	0.512	4.728	0.436
F2 Future service contact	0.686	0.513	0.859	0.050	0.407	0.167	0.990	0.047
F3 Future response to drug treatment	0.686	0.513	0.859	0.050	0.407	0.167	0.990	0.047
F4 Future response to psychological intervention	0.729	0.579	0.879	0.016	0.323	0.129	0.809	0.016
F5 Future Stress	0.520	0.328	0.712	0.831	0.803	0.264	2.444	0.699

Note that lower scores are calculated as positive predictors of discharge yielding higher AUCs. *AUC* area under the curve (receiver operating characteristic). ‘p’ = Asymptotic probability for null hypothesis that AUC = 0.5. Unadjusted odds ratios, note that some ORs could not be calculated.

The PANSS symptoms that were associated with subsequently not being discharged were P1 ‘delusions’, P6 ‘suspiciousness / persecution’, N1 ‘blunted affect’, N4 ‘passive/apathetic/social withdrawal’, N5 ‘difficulty in abstract thinking’ and G12 ‘lack of judgement and insight’ (Table 10).

**Table 10:** PANSS items and AUC for discharge

**PANSS items**	**Area Under Curve (AUC)**	**95% confidence interval of AUC**		**p**	**Odds Ratio (OR)**	**95% confidence interval of OR**		**p**
		**Lower**	**Upper**			**Lower**	**Upper**	
P1 delusions	0.690	0.536	0.884	0.046	0.676	0.540	0.845	0.006
P2 conceptual disorganisation	0.628	0.647	0.785	0.179	0.676	0.540	0.845	0.006
P3 hallucinations	0.547	0.371	0.722	0.625	0.739	0.623	0.878	0.020
P4 hyperactivity	0.566	0.392	0.739	0.488	0.905	0.645	1.268	0.608
P5 grandiosity	0.528	0.348	0.708	0.768	0.745	0.630	0.880	0.029
P6 suspiciousness / persecution	0.721	0.581	0.861	0.020	0.714	0.590	0.865	0.005
P7 hostility	0.641	0.485	0.798	0.137	0.831	0.647	1.067	0.270
N1 blunted affect	0.735	0.591	0.880	0.013	0.720	0.564	0.919	0.010
N2 emotional withdrawal	0.675	0.501	0.849	0.065	0.736	0.563	0.964	0.022
N3 poor rapport	0.672	0.527	0.818	0.070	0.773	0.615	0.972	0.056
N4 passive / apathetic, social withdrawal	0.725	0.582	0.867	0.018	0.720	0.564	0.919	0.010
N5 difficulty in abstract thinking	0.729	0.568	0.889	0.016	0.586	0.432	0.796	0.001
N6 lack of spontaneity and flow of conversation	0.680	0.524	0.836	0.058	0.700	0.571	0.857	0.002
N7 stereotyped thinking	0.534	0.350	0.718	0.721	0.707	0.581	0.861	0.003
G1 somatic concern	0.543	0.366	0.719	0.654	0.905	0.645	1.268	0.608
G2 anxiety	0.448	0.273	0.622	0.582	0.816	0.636	1.047	0.136
G3 guilt feelings	0.448	0.259	0.637	0.582	1.107	0.827	1.481	0.481
G4 tension	0.448	0.306	0.671	0.903	0.714	0.590	0.865	0.005
G5 mannerisms and posturing	0.529	0.348	0.710	0.760	0.765	0.675	0.890	0.111
G6 depression	0.547	0.372	0.721	0.625	0.905	0.645	1.268	0.608
G7 motor retardation	0.576	0.403	0.748	0.427	0.733	0.615	0.875	0.014
G8 uncooperativeness	0.644	0.490	0.799	0.129	0.881	0.650	1.194	0.484
G9 unusual thought content	0.683	0.535	0.832	0.054	0.721	0.599	0.868	0.007
G10 disorientation	0.535	0.356	0.714	0.714	0.778	0.674	0.897	0.321
G11 poor attention	0.601	0.433	0.768	0.289	0.684	0.551	0.849	0.001
G12 lack of judgement and insight	0.784	0.639	0.929	0.003	0.600	0.448	0.804	0.001
G13 disturbance of volition	0.581	0.412	0.750	0.392	0.721	0.599	0.868	0.007
G14 poor impulse control	0.604	0.439	0.769	0.276	0.784	0.623	0.986	0.076
G15 preoccupation	0.570	0.402	0.737	0.463	0.692	0.562	0.853	0.002
G16 active social withdrawal	0.591	0.421	0.761	0.338	0.802	0.625	1.030	0.104

Area under the curve (AUC) and unadjusted odds ratio (OR) that higher score reduces probability of discharge. OR is the odds ratio that increased score reduces probability of discharge.

Table 11 shows the 61 items from all of the above scales and instruments with confidence intervals for the AUC that did not overlap the random value of 0.5, arranged in order of magnitude of the AUC. A few themes appear to emerge including global function and coping skills, insight and mental state, problem behaviours and specialist forensic need, negative symptoms, therapeutic rapport, substance misuse problems, victim issues and physical health.

**Table 11:** Variables in order of magnitude of area under the curve for prediction of discharge

**Variable**	**AUC**
GAF	0.930
D3 PC5 Self care and activities of daily living	0.881
START-strength S17 9(s) Insight	0.873
D4 R2 Insight	0.868
D3 PC4 Problem behaviours	0.865
D3 PC1 Physical health	0.856
D3 PC2 Mental health	0.855
D4 R1 Stability	0.851
D3 PC6 Education, occupation and creativity	0.850
D1 TS5 Specialist forensic need	0.845
START S17 (v) Insight	0.835
START S19 (s) Coping	0.830
START S6 (s) Mental state	0.828
START S2 (s) Relationships	0.818
D4 R5 Dynamic risk items	0.805
START ST18 (v). Plans	0.795
START S19 (v) Coping	0.788
SAPROF S11 Life goals	0.786
HCR-20 C1 Lack of insight	0.779
START ST18 Plans (s)	0.773
START ST20 Treatability (v)	0.763
SAPROF SO13. Social Network	0.761
HCR-20 C5 Unresponsiveness to treatment	0.760
HCR-20 H3 Relationship instability	0.749
D4 R6 Victim sensitivity	0.748
HCR-20 H4 Employment problems	0.745
D3 PC3 Drugs and alcohol	0.742
START ST6 (v) Mental state	0.739
START ST2 (v) Relationships	0.736
PANSS N1 Blunted affect	0.735
SRAMM F4 future response to psychological intervention	0.729
PANSS N5 Difficulty in abstract thinking	0.729
START ST11 (v) Social support	0.727
START ST3 (v) Occupational	0.727
D1 TS7 Preventing access	0.726
PANSS N4 Passive/apathetic/social withdrawal	0.725
START ST1 (v) Social skills	0.723
HCR-20 C3 Active symptoms of major mental illness	0.722
SAPROF SO4 Coping	0.721
PANSS P6 Suspiciousness/ persecution	0.721
D4 R3 Therapeutic rapport	0.720
HCR-20 R1 Plans lack feasibility	0.719
START ST10 (s) External triggers	0.714
D4 R4 Leave	0.711
SRAMM SC7 Psychosocial stress	0.709
SAPROF SO8 Financial management	0.705
D3 PC7 Family and social networks	0.697
START ST3 (s) Occupational	0.696
PANSS P1 Delusions	0.690
SAPROF SO5 Self control	0.686
SRAMM SC4 Treatment adherence	0.686
SRAMM F2 Future service contact	0.686
SRAMM F3 Future response to drug treatment	0.686
PANSS G9 Unusual thought content	0.683
SRAMM SC8 Problem solving deficits	0.682
PANSS N6 Lack of spontaneity and flow of conversation	0.680
SAPROF SO6 Work	0.678
START ST1 (s) Social skills	0.678
START ST7 (s) Emotional state	0.678
PANSS N2 Emotional withdrawal	0.675
START ST7 (v) Emotional state	0.675

*GAF* global assessment of functioning, *D3* DUNDRUM-3 programme completion, *D4* DUNDRUM-4 recovery.

## Discussion

### Key results

This was not an actuarial study. This study was not primarily about predicting discharge though this forms one part of validation. The main purpose was to validate the content of structured professional judgement instruments to aid decision making regarding discharge from a forensic hospital. The MHRBs were blind to the instrumental ratings made prior to the commencement of the new legislation. They were not blind to the clinical facts of the individual patient’s progress. The object of the study was to examine whether the content of the instruments captured the clinical information that influenced the decision makers. A successful validation would be evidence from receiver operating characteristics and in regression models that the instruments accounted for most of the outcomes. This proved to be the case. We believe the study demonstrated that simple risk assessments account for only some of the outcome, with treatment completion and recovery measures also contributing. Further, we wanted to examine whether all items in these structured professional judgement instruments were relevant. We found that some instruments that had scale scores associated with conditional discharge had only a few items that were relevant to this outcome, though other items might be relevant to other outcomes such as later reoffending or recall to hospital. Odds ratios also demonstrated that not all items were equally influential, some having larger effects (stronger associations with outcomes) than others.

This study occurred as a result of an experiment of nature. In February 2011, legislation was introduced for the first time in the Republic of Ireland, granting to the Mental Health Review Board the power to grant conditional discharge to patients who were detained in a forensic hospital having been found unfit to stand trial or not guilty by reason of insanity. We were therefore able to test the relevance of the content of structured professional judgement instruments assessing need for therapeutic security, risk assessment, assessment of progress in treatment, recovery, symptoms and global function by assessing these prior to the first hearings under the new legislation. During the period of observation, the DUNDRUM-3 programme completion and the DUNDRUM-4 recovery scales distinguished which patients later received a conditional discharge. The DUNDRUM-1 assessment of need for therapeutic security - a static measure - did not distinguish subsequent conditional discharge apart from a low score on ‘specialist forensic need’, an item describing arson, sadism, paraphilias and lack of cooperation. Low scores on the HCR-20 scales for risk of violence also distinguished which patients received a conditional discharge, as did low scores on the S-RAMM for assessing risk of self-harm and suicide. High (better) scores for the Global Assessment of Function (GAF) had a better AUC than any of the risk assessment scales. Low scores on the PANSS scales for positive, negative and general symptoms also distinguished subsequent conditional discharge.

Co-varying for the HCR-20 score left only the DUNDRUM-3 and DUNDRUM-4 as significantly associated with conditional discharge, and the DUNDRUM-4 was eliminated when its risk item was omitted. However when the HCR-20 scales were co-varied for either the DUNDRUM-3 or the DUNDRUM-4 (omitting its risk item) the HCR-20 was no longer statistically significant for conditionally discharged patients.

It is particularly interesting that the Global Assessment of Function (GAF) emerged as one of the strongest precursors of conditional discharge, and dominated the logistic regression models. The DUNDRUM-3 and DUNDRUM-4 correlated strongly with the GAF (Spearman r for DUNDRUM-3 and GAF, r = −0.804, p < 0.001, DUNDRUM-4 and GAF r = −0.729, p < 0.001) and the DUNDRUM-3 programme completion scale emerged as the next strongest statistical precursor. It is possible that the items of these scales represent elements of what is taken into account when rating the GAF, or it may be that an element in the rating of all of these items, like the GAF, is a rating of some ‘structured intuition’ as recently suggested [27]. The ratings of the seven items of the DUNDRUM-3 programme completion scale and the six items of the DUNDRUM-4 recovery scale have in common that they are based on motivation theory, cycle of change and engagement.

On an item to outcome analysis all items of the DUNDRUM-3 programme completion scale and of the DUNDRUM-4 recovery scale significantly predicted conditional discharge while five of the HCR-20 items, four of the SAPROF items, seven of the START-S items, nine of the START-V items, five of the S-RAMM items and six PANSS items contributed to the statistical relationship with the outcome.

### Limitations

The ratings of the instruments and their component items were carried out by researchers who were blind to each others’ ratings and the research ratings were not provided to the clinicians or MHRBs. The clinicians and MHRB were therefore blind to the baseline ratings. The success or failure of the instruments as statistical predictors depends on the extent to which they emulate the ways in which the clinicians and MHRBs make their recommendations and decisions. The clinical information contained in the structured professional judgement instruments was inevitably available in unstructured form to the clinicians and MHRBs who could not be deprived of it. What was studied is the extent to which the structured instruments and their contents at baseline, were associated with the later behaviour of the decision makers. The statistical inference is that the decision makers relied to a greater or lesser extent on this information (in unstructured form). This was an observational study and therefore no special effort could be made to blind clinicians who gave evidence or the members of the MHRB to such unstructured information. Since the purpose of the study was to identify in a defined structured form those clinical facts that influence conditional discharge, it would be self-defeating and probably impossible to blind clinicians and MHRB members to such unstructured information.

This study did not attempt to distinguish between the recommendations of the treating psychiatrists and the decisions of the mental health review board (MHRB) since that would have required greater numbers and access to the deliberations of a judicial body.

A further limitation of this study is that many of the measures are dynamic. Ratings made up to 18 months before a hearing may therefore be attenuated by subsequent change. Significant associations (receiver operating characteristics, odds ratios) are therefore conservative estimates.

This was a study of factors influencing the decision by a mental health review board (MHRB) to conditionally discharge, it was not a study of adverse outcomes following discharge. One of the weaknesses of this study is that a number of patients had been considered ready for conditional discharge for some time prior to this being introduced into Irish legislation. Because these patients had been delayed so long, many of the patients achieving conditional discharge had near perfect scores on the DUNDRUM-3 and DUNDRUM-4 scales. We believe this problem has to an extent been overcome by the period of follow-up, examining conditional discharges after three MHRB hearings rather than just the first hearing under new legislation. In the future patients may be offered a conditional discharge with less perfect scores.

### Interpretation

This is a study of the overall process by which clinicians make recommendations and MHRB members decide who to discharge. We have shown that clinicians and decision makers appear to take into account more than risk alone when deciding to release a patient from a secure hospital setting.

A thematic analysis of the items listed in Table 11 suggests that amongst the strongest statistical discriminants of subsequent discharge are items specifically related to societal considerations such as victim sensitivity, psychosocial stress and family and social networks. A second prominent theme concerns rehabilitation and recovery items such as stability, global function, insight, self care, occupation, negative symptoms and mental health. A third theme could be summarised as features of offending such as problem behaviours, dynamic risk, specialist forensic need and early maladjustment . Indicators of good working relationships with clinicians such as responsiveness to treatment, therapeutic rapport and use of leave outside the hospital (trust) also formed a significant theme.

The handbook of the DUNDRUM toolkit [10] emphasises the importance of motivation for change and fulfilment of personal needs, as well as working alliance and interpersonal trust in clinicians [28]. It may be that these ‘higher order’ themes account for the apparent success of the DUNDRUM-3 treatment completion and DUNDRUM-4 recovery scales in predicting which patients clinicians will recommend for conditional discharge and who the members of the MHRB will decide should be discharged.

### Generalisability

The DUNDRUM-3 programme completion and the DUNDRUM-4 recovery scales appear to predict moves to the community from the forensic hospital setting. In another naturalistic prospective cohort study in the same population but a year earlier we reported that the DUNDRUM-1 triage security scale and the HCR-20 measure of risk of violence predicted which patients would be moved from more secure to less secure wards within the hospital, and who would be moved back from less secure to more secure wards [14]. The HCR-20 has been shown to predict those who have adverse outcomes on discharge from forensic hospitals [5-7]. Future research will be required to assess whether or not the DUNDRUM-3 programme completion and the DUNDRUM-4 recovery scales can predict those patients who will succeed in the community setting and those patients who will require to be recalled to the forensic hospital setting. It may be that different sets of items within the various structured professional judgement instruments and symptom inventories will predict violent recidivism, relapse or readmission. Those items that appear to be ‘inactive’ in this study may prove more important in the community. A follow up study over a prolonged period will be necessary to examine that question. We believe that a multi-centre, international study may be needed to achieve statistical power for a sufficiently detailed analysis.

The development of structured professional judgement instruments to assist decision making in areas other than risk assessment should be of broad interest to clinicians other than forensic psychiatrists. The decision to discharge any in-patient is among the most professionally challenging in any medical or psychiatric setting, accompanied as it is by risks of subsequent self neglect, relapse, social and environmental hazards, suicide and in rare cases harm to others. The efforts here to describe this decision making process, leading to future validation of those factors other than risk that might be associated with positive or adverse outcomes, is of benefit for a range of applications. Such structured professional judgement may be a way of containing naturally occurring bias in decision making [29]. Medical educators may find this useful as well as those concerned with decision making in mental health tribunals and review boards, the commissioners of services and service managers who plan treatment programmes and aftercare.

## Conclusions

The DUNDRUM-3 and DUNDRUM-4 structured professional judgement instruments were designed to compliment risk assessment instruments as a means of guiding the clinicians and decision makers concerning those who are ready for the move from secure forensic hospitals to the community. In this naturalistic observational study of a cohort of patients with measured needs for therapeutic security at medium secure level (using the DUNDRUM-1 triage security measure), the DUNDRUM-3 and DUNDRUM-4 appear to capture information relevant to the decision to discharge, adding significantly to the information gained from risk assessment alone. Whether they also predict those who are not recalled or do not reoffend after conditional discharge remains to be studied.

## Competing interests

The authors declare that they have no competing interests.

## Authors’ contributions

MD rated patients using the DUNDRUM-1, DUNDRUM-3 and DUNDRUM-4, revised drafts of the article and collated the database with ZA; LN and OG rated patients using the PANSS and GAF. The HCR-20 and S-RAMM were rated by the treating clinicians and collated by AN. ZA rated all patients using the START and SAPROF. HGK wrote the first draft of the handbook, designed the study and carried out the data analysis with MD. All contributed to the authorship of the paper. All authors read and approved the final manuscript.

## Pre-publication history

The pre-publication history for this paper can be accessed here:

http://www.biomedcentral.com/1471-244X/13/185/prepub
